# Repeatability of the resonance frequency analysis values in implants with a new technology

**DOI:** 10.4317/medoral.22761

**Published:** 2019-08-18

**Authors:** Mª Carmen Díaz-Castro, Artur Falcao, Paula López-Jarana, Carlos Falcao, Jose-Vicente Ríos-Santos, Ana Fernández-Palacín, Mariano Herrero-Climent

**Affiliations:** 1 PhD, DDS, MSc (Periodontics). Clinical Teaching Fellow in the Master’s Program of Periodontology and Implant Dentistry from University of Seville (Spain); 2 MSc. Clinical Lecturer. Fernando Pessoa University and Porto Dental Institute, Porto. (Portugal); 3 DDS, MSc, PhD. Clinical Lecturer Fernando Pessoa University and Porto Dental Institute, Porto. (Portugal); 4 PhD, MD, DDS. Clinical Lecturer, Department of Periodontics and Implant Dentistry, Co-director Master’s Degree in Periodontics and Implant Dentistry, Dental School University of Seville (Spain); 5 PhD, Department of Biostatistic, Dental School University of Seville (Spain); 6 MD, DDS MSc, PhD. Fernando Pessoa University and Porto Dental Institute, Porto. (Portugal)

## Abstract

**Background:**

Assess the reliability (by means of reproducibility and repeatability) of the PenguinRFA system, analyse the ISQ values of different implant types and correlate the ISQ with the insertion torque during the placement of the implant.

**Material and Methods:**

120 rough surface implants were placed in bovine bone (type II and III). The implants were divided into groups, according to its design. Once the implants were in place, the exact insertion torque was registered. Then, primary stability was measured by means of the resonance frequency analysis with the PenguinRFA and the Osstell ISQ devices. In each implant two transducers of each device were used. Three measurements were obtained with each transducer.

**Results:**

The mean ISQ (implant stability quotient) of the whole sample is 67,70 ± 5,51. The Intraclass Correlation Coefficient (ICC) is 0,933 and 0,944 for transducers 1 and 2 respectively. The reproducibility is 0,906. The mean insertion torque is 24,54 ± 8,96N. The correlation between the ISQ and the insertion torque is 0,507 *p*<0,000 (MultiPeg 1) and 0,468 *p*<0,000 (MultiPeg 2) for bone type II and 0,533 *p*<0,801 (MultiPeg 1) and 0,193 *p*<0,140 (MultiPeg 2) for bone type III.

**Conclusions:**

The results of the present trial suggest that the PenguinRFA presents excellent reproducibility and repeatability, so it could be very useful in the monitoring of the stability of implants over time. Additionally, according to the results, the correlation between the IT and the RFA is low and there are no statistically significant differences in between implant types.

** Key words:**Implant stability, insertion torque, ISQ, osseointegration, implant-supported dental prostheses, immediate dental implant loading.

## Introduction

Substitution of missing teeth with dental implants represents one of the most successful treatment modalities in dentistry. The growing interest in the management of soft tissues and in the improvement of patient comfort leads us to load the implants immediately. Although the literature reports similar success rates for delayed implants and immediate implants, the latest technique needs some requirements, the most important being the primary stability.

Primary implant stability (the absence of mobility in bone site after implant insertion) is essential for adequate implant osseointegration (1-3). Secondary stability results after the formation of woven and lamellar bone around the dental implant surface as a secondary bone contact (1,2). The maintenance of adequate stability over time is also considered a long-term success guarantee (3,4). Some studies have shown that extensive micromotion during healing and loading could be the reason for implant failure, as this may result in a non-mechanical connection between the implant surface and the surrounding bone. The greater the primary stability, the smaller the micromotions between implant and bone (5).

Resonance Frequency Analysis (RFA) represents a non-invasive method for clinical assessment of implant stability. It was introduced by Meredith in 1996 and is an extensively used tool for an impartial estimation of implant stability at any phase of treatment or follow-up due to its high reliability and reproducibility. It enables the clinician to monitor the stability of the implant over time and analyse its evolution (6-8). The unit of measure of RFA is the implant stability quotient (ISQ), and its scale values can oscillate from 1 to 100. The higher the ISQ number, the higher the stability (3). Moreover, Pagliani has reported that RFA measurements can be correlated with the micromobility of dental implants, as this micromobility seems to be definite by the bone density at the implant site (9). It is known that several factors can affect the ISQ values (10), such as the effective implant length; the distance from the transducer to the marginal bone (the greater the distance from the transducer to the bone, the lower the ISQ value) (3,11); the osseous quality (12); the strength with which the transducer is torqued (11,13); the existence of soft tissue between the implant and the transducer (12,13); and the quantity of bone in contact with the implant (12).

Recently, a new generation of RFA technology has been developed. The new system consists of a small pen-like battery-driven instrument (PenguinRFA), and the transducers (MulTiPegTM) are reusable (they could be autoclaved and used numerous times) because they are made of titanium (they do not lose the capacity to be tightened several times, unlike those made of aluminium). The ISQ values are shown in two screens, one each side of the device.

Currently, different RFA technologies coexist and the ISQ values during monitoring of stability can be taken with different apparatus and transducers. That being so, it is necessary to know if the measurements are comparable.

On the other hand, insertion torque (IT) (the moment of force necessary to seat the implant into the osteotomy site) (14) is another widely used method for evaluating the primary stability. The determination of the IT is done by a torque gauge incorporated into the drilling unit or with a torque wrench during the insertion of the implant (15,16). It is an easy method, but it can be measured only once, when the implant is placing.

Many reports have tried to clarify whether ISQ and IT are correlated. Some studies have shown that ISQ provides information on axial stability, whereas IT measures rotational stability (17). Additionally, the correlation of higher IT to greater primary stability may not always be true because the quantity and quality of bone varies significantly among patients (18).

The aim of the present trial was to assess the reliability (by means of reproducibility and repeatability) of the PenguinRFA system, analyse the ISQ values of different implant types and correlate the ISQ with the insertion torque during the placement of the implant.

## Material and Methods

The study was carried out at the Porto Dental Institute.

**Figure 1:**
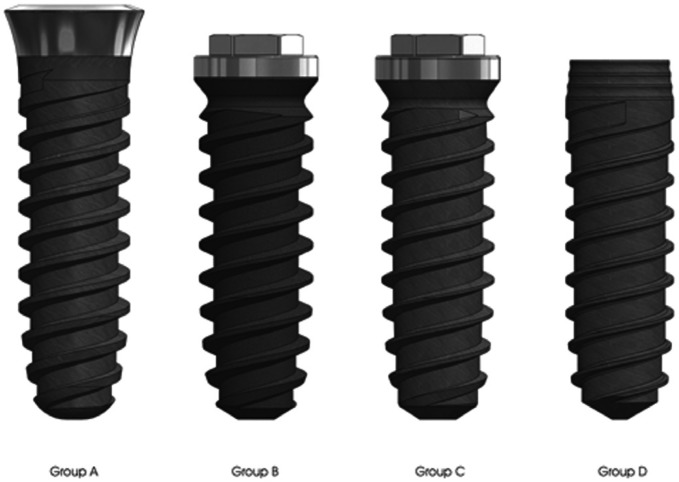
Implants used in the study.

120 rough surface implants (Shot Blasting®: alumina particle sandblasting and acid passivation) screw-shaped implants (Klockner Implant System, SOADCO, Andorra) were employed. The implants were divided into one of the following groups (Fig. 1):

Group A: 30 Essential Cone Implants (12mm long and 3,5 mm in diameter). These are internal connection, double-threaded implants, characterized by an atraumatic apex, a progressive core and a machined collar.

Group B: 30 KL standard implants implants (12mm long and 3,75 mm in diameter). These are external connection, double-threaded implants, characterized by a self-tapping apex and a progressive core. They have an external hexagon and are compatible with Branemark implants.

Group C: 30 KL prototype implants (12mm long and 3,75 mm in diameter). These are the same as the standard implants, but the progressive core is 0,2 mm wider.

Group D: 30 VEGA Implants (12mm long and 3,5 mm in diameter). These are internal connection, double-threaded implants, characterized by an atraumatic apex and a progressive core. They are designed to be placed at a crestal level.

The implants were inserted in bovine bone, by an experienced clinician (user of the Klockner Implant System for more than 2 years). Fifteen implants of each group were placed in bone quality type II (bovine ribs), and the other fifteen were placed in bone quality type III (bovine femoral epiphysis), according to Lekholm & Zarb (19). The ribs and epiphysis were selected from the same animal, assessing that they were of the same size as the only inclusion criteria. To ensure a balance in sample size, the block randomization method is used. To be sure that the number of implants included in each group is the same, a balanced randomization was carried out in eight blocks (group A, B, C, D combined with bone type II, type III). The 120 closed envelopes, contained each possibility, were opened before site preparation. The implant site preparation was carried out under generous irrigation with sterile saline solution, following the manufacturer’s instructions for each implant type. A manual torque wrench was used to place the implants, so the exact insertion torque was registered. The implants were inserted until the rough/smooth interface was at the bone crest level in groups A, B and C. In group D, the implant was placed at the crestal level. The implants should be placed, at least, 4 mm apart.

After implant placement, primary stability was registered with the PenguinRFA and Osstell ISQ devices by a second experienced clinician, following the manufacturer instructions. The transducers were screwed by the specific hand-screwdriver (approximately 6-8 Ncm of torque) to the implant and the ISQ was registered. The RFA was measured perpendicular to the transducer, approximately 2 mm from it. In all cases, the ISQ was registered from the front of the rib or the epiphysis. In each implant, two MultiPegs and two Smartpegs (transducers) were used. Three measurements were obtained with each transducer; in between them, the transducer was unscrewed and screwed again.

SPSS 19,0 software (SPSS, Chicago, IL) was employed for the statistical analysis. Mean values and standard deviations were calculated. The normal distribution of the values and the homogeneity of the variances were confirmed through Kolmogorov-Smirnov and Levene tests, respectively. The differences between the mean values were analysed with the non-parametric Kruskal-Wallis and Mann-Whitney tests. When significant differences appeared, 95% confidence intervals were found for average and mean differences (*p*<0,05). The Intraclass Correlation Coefficients (ICC) were calculated to study the concordance between consecutive measurements by the same device on the same implant. Finally, to analyse the correlation between ISQ and IT the Pearson Correlation was also obtained.

## Results

The mean ISQ of the 120 implants is 67,70±5,51. The mean ISQ of the two devices used are shown in  Table 1. The mean ISQ for PenguinRFA is 67,70±5,51 and for Osstell ISQ is 68,55±9,03. The ICC between PenguinRFA and Osstell ISQ is 0,77 (0,67-0,84; *p*< 0,0005).

**Table 1:**
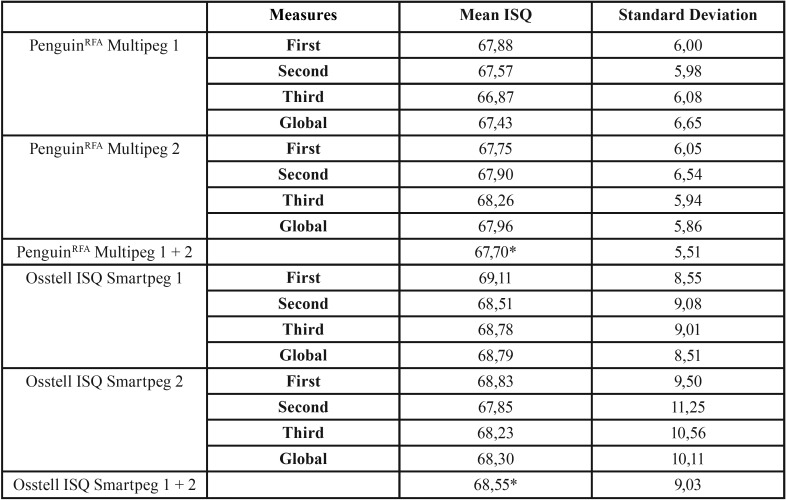
ISQ (implant stability quotient) values (mean and standard deviation) of the whole sample. * have statistically significant differences between them. *p*<0,0005.

The mean ISQ for Essential, Standard, Prototype and VEGA implants are 68,99±4,67, 65,59±5,41, 68,03±7,47 and 68,19±3,31, respectively.  Table 2 displays the ISQ values according to the type of implant and transducer used, when the PenguinRFA was employed.

**Table 2:**
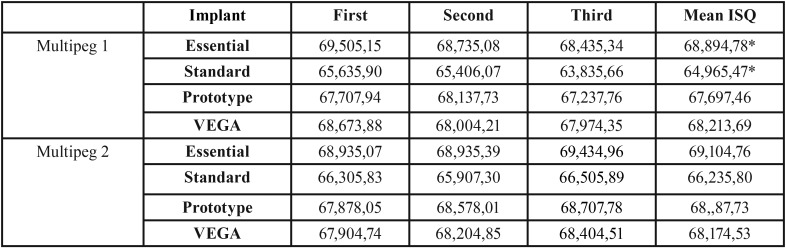
ISQ (implant stability quotient) values (mean and standard deviation), obtained by de PenguinRFA according to the type of implant and transducer used. No statistically significant differences were found (p=0.18), except for Multipeg 1 in between Essential and KL standard implants (* *p*=0.023).

Regarding the reliability of the Penguin, the intraclass correlation coefficient (ICC) is 0,933 and 0,944 for MultiPegs 1 and 2 respectively, with a confidence interval at 95%. This indicates an almost-perfect degree of concordance between PenguinRFA transducers. Subsequently, MultiPeg 1 and 2 measurements are compared to assess differences among the six completed measurements, so the reproducibility is 0,906. The ICCs according to the different types of implants are given in  Table 3. All the values showed with a *p*<0,005 except for the ICC of the PenguinRFA when the VEGA implant is analysed, (*) *p*>0,05.

**Table 3:**
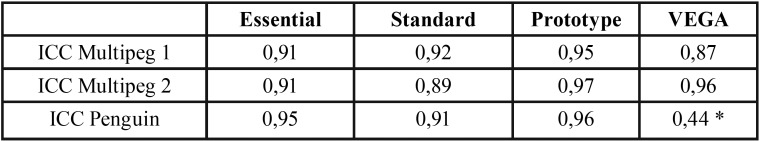
The ICC according to the different types of implants. (*) *P* > 0,005. ICC: Intraclass Correlation Coefficient.

The analysis of the ISQ based on the quality of bone shows that the ISQ for bone type II is higher than for bone type III. For type II bone the ISQ is 68,46±4,69 and 68,74±4,96 when the RFA is measured over the MultiPeg 1 and 2 respectively (*p*= 0,079). For type III bone, the ISQ is 68,74±4,96 and 67,19±6,60 when Multipeg 1 and 2 are used (*p*=0,244). The differences are not statistically significant. The ICC is 0,97 and 0,95 (*p*<0,0001) for bone type II; and 0,91 and 0,94 for bone type III, when Multipeg 1 and 2 are used respectively.

The mean insertion torque (IT) of the entire sample is 24,54±8,96 Ncm. The mean IT of implants placed in bone type II is 26,85±8,57 Ncm and in type III is 22,23±8,80 Ncm. According to the type of implants, the IT is 24,73±9,48 Ncm for Essential implants. 25,60±9,74 Ncm for KL standard implants, 28,73±7,99 Ncm for KL prototype implants and 19,19±5,51 Ncm for VEGA implants. The differences are only statistically significant between the VEGA implants and each ot the other groups (*p*<0,0005).

The correlation between the IT and the FRA measured with the PenguinRFA device and the MultiPeg transducer is 31. The correlations studied by implant types are given in  Table 4.

**Table 4:**
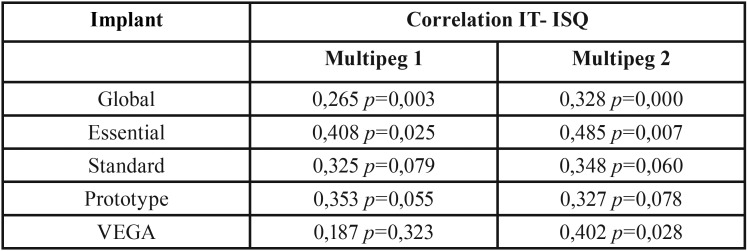
Pearson’s Correlations between insertion torque and ISQ analyzed by implant type. IT: insertion torque; ISQ: implant stability quotient.

The correlation between the ISQ and the IT is 0,507 *p*<0,000 (MultiPeg 1) and 0,468 *p*<0,000 (MultiPeg 2) for bone type II and 0,533 *p*<0,801 (MultiPeg 1) and 0,193 *p*<0,140 (MultiPeg 2) for bone type III.

## Discussion

The mean ISQ of the implants included in the study is 67,70±5,51. This ISQ is lower than those of similar studies that use cow rib models (20,21). The slight differences between the results of those trials and the present trial could be due to the different macro design of the implant used. Analysing the literature, it seems that the endosseous screw-shaped implant were the most suitable design. In 1999, it was found that the 8 mm implants reached the higher primary stability (22). These implants were all inserted in the posterior mandible, where bone quality type II was observed. Fu et al. (23) found that the ISQ value was slightly related to the bone type when evaluated by stereomicroscopy or micro-CT in the upper jaw.

The ISQ values obtained with the two devices (Osstell ISQ and PenguinRFA) show differences statistically significant. These differences are only of less than one point (68,55±9,03 vs 67,70±5,51), which could have no clinical significance. Although the slightly differences, the reliability between the two devices is good, as the ICC is 0,77.

Regarding the different types of implants ( Table 2), the Essential Cone implants are the implants that reach the higher ISQ. In contrast, the KL Standard ones are the implants with the lower ISQ values. In the literature, some publications compared the ISQ between implants placed at different height with regard of the crest level, and the results they obtained are not consistent. Dursun *et al.*, in 2012, (24) showed that mean ISQ values for the bone level implants were lower than for the tissue level implants at the time of placement. These results are in agreement with those of the present trial. On the other hand, Anil *et al.*, in 2015, (25) found that implants placed, also in bovine ribs, at the crestal level showed slightly higher but insignificant ISQ values when compared to the tissue level implants. Coutant *et al.* (26) observed variations in primary stabilities depending on the implant design.

Reliability is defined as the extent to which measurements can be replicated. In this study, the reliability is measured by means of repeatability (several attempts with the same transducer lead to similar results), and reproducibility (different transducers on the same implant provide similar data). The repeatability and the reproducibility of the PenguinRFA device is excellent as the ICC are over 0,90

To our knowledge, this is the first study assessing the reliability of the PenguinRFA. However, the results are similar to trials studying the reliability of other RFA systems. In 2013, Herrero-Climent *et al.* (7) found that the repeatability and reproducibility for the Osstell ISQ are 0,97. Geckili *et al.* (27), in 2012, showed that there were no differences between the RFA measurements of the Osstell Mentor and Ostell ISQ (but if the measurements are no made by the same examiner the concordance is poor). In 2014, Jaramillo *et al.* (8) reported an almost perfect degree of concordance between the Osstell Mentor and the Osstell ISQ (ICC=0,98).

The repeatability according to the implant type is also quite high (all values are over 0,75). However, the reliability assessed for the VEGA implant is moderate. For VEGA implants, the repeatability was high but the reproducibility was not. This would mean that the measures are repeatable when the same transducer is used, but not when the transducers are changed. The explanation for those results could be because for 2 implants, the values obtained from one of the MultiPegs were lower than the ones for the rest of the sample. The other types of implants did not follow that trend and show excellent reproducibility.

Pearson’s correlation coefficient showed that the correlation between the IT and the RFA is positive, but low. The results are in agreement with those published by Degidi *et al.* (15,16) and Makari *et al.* (28). In contrast, some other trials (1,2,17,29) show a higher correlation between the IT and the ISQ values. The differences between these studies and ours could be due to different factors: all of their implants were conducted in human patients, and in some of them, the insertion torque was recorded by an electric motor. Additionally, Brizuela-Velasco *et al.* (30) reported a direct correlation between insertion torque and ISQ values under a load of 100 N. They also found an inverse correlation between micromotion and ISQ and IT; however, in their case, the IT inverse correlation was exponential, and the ISQ inverse correlation was linear.

The results of the present trial suggest that the PenguinRFA presents excellent reproducibility and repeatability, so it could be very useful in the monitorization of the stability of implants over time. Additionally, according to the results, the correlation between the IT and the RFA is low and there are no statistically significant differences in between implant types.
